# Apoptosis and frequency of total and effector CD8^+^ T lymphocytes from cutaneous leishmaniasis patients during antimonial therapy

**DOI:** 10.1186/s12879-015-0799-x

**Published:** 2015-02-19

**Authors:** Raquel Ferraz, Clarissa F Cunha, Adriano Gomes-Silva, Armando O Schubach, Maria Inês F Pimentel, Marcelo Rosandiski Lyra, Sergio CF Mendonça, Cláudia M Valete-Rosalino, Alda Maria Da-Cruz, Álvaro Luiz Bertho

**Affiliations:** Laboratory of Immunoparasitology, Oswaldo Cruz Institute, FIOCRUZ, Rio de Janeiro, RJ Brazil; Flow Cytometry Sorting Core, Oswaldo Cruz Institute, FIOCRUZ, Rio de Janeiro, RJ Brazil; Laboratory of Interdisciplinary Medical Research, Oswaldo Cruz Institute, FIOCRUZ, Rio de Janeiro, RJ Brazil; Laboratory of Surveillance for Leishmaniasis, Evandro Chagas National Infectology Institute, FIOCRUZ, Rio de Janeiro, RJ Brazil; Department of Otolaryngology and Ophthalmology, Medicine College, Federal University of Rio de Janeiro, Rio de Janeiro, RJ Brazil; Laboratory of Immunoparasitology and Flow Cytometry Sorting Core, Oswaldo Cruz Institute, FIOCRUZ, Av. Brasil, 4365, Manguinhos, Pavilhão Leônidas Deane, sala 408-A, CEP: 21040-900 Rio de Janeiro, RJ Brasil

**Keywords:** Flow cytometry, Effector CD8^+^ T lymphocytes, Apoptosis, Human cutaneous leishmaniasis, During antimonial treatment, *Leishmania braziliensis*

## Abstract

**Background:**

Leishmaniasis is an important parasitic disease affecting millions worldwide. Human cutaneous leishmaniasis (CL) is endemic in Rio de Janeiro, Brazil, where is caused by *Leishmania braziliensis*. The adaptive immune response is accountable for the healing of CL and despite of key role of CD8^+^ T cells in this immune response little is known about the CD8^+^ T lymphocytes frequencies, apoptosis and antigen-responsive CD8^+^ T lymphocytes of CL patients during antimonial therapy.

**Methods:**

Using flow cytometry, we examined total and effector CD8^+^ T cells from CL patients before (PBT), during (PDT) and after (PAT) treatment for apoptosis and frequencies upon isolation and after *in vitro L. braziliensis* antigens (LbAg)-stimulation culture. Besides, a correlation study between immunological findings and lesion size was done.

**Results:**

PDT showed lower frequencies of total CD8^+^ T lymphocytes and higher levels of apoptosis of these cells, which were also observed following LbAg-stimulation culture. Regarding effector CD8^+^ T cells, high frequencies were observed in PDT, while lower frequencies were observed in PAT. Interestingly, PDT showed higher frequencies of apoptotic-effector CD8^+^ T lymphocytes. Similar results were seen after *in vitro* antigenic-stimulation assays. Correlation analysis showed that the greater the size of lesion, the smaller the frequency of effector CD8^+^ T lymphocytes in PDT and PAT, as well as a positive correlation between apoptotic-effector CD8^+^ T cells frequency and lesion size of PDT.

**Conclusions:**

Changes in effector CD8^+^ T–lymphocyte frequencies, during and after treatment, seem to represent a critical stage to generate an efficient immune response and suggest that these cells would be evolved in the triggering or in the resolution of lesion, under the influence of therapy. This hypothesis opens new perspectives to clarify controversial statements about the protective or deleterious role of CD8^+^ T cells in the cure or aggravation of CL and the new approach of evaluating patients during treatment proved to be of utmost importance for understanding the immune response in the healing process of human CL.

## Background

Leishmaniasis is a group of diseases caused by different species of protozoan parasites from the genus *Leishmania* and is ranked as the sixth major neglected tropical disease in the world. In Brazil, American tegumentary leishmaniasis (ATL) was registered in all states and is endemic in Rio de Janeiro, where it is caused mainly by *Leishmania (Viannia) braziliensis*, leading to a spectrum of clinical, immunological and histopathological manifestations, ranging from self-healing localized cutaneous leishmaniasis (CL) to destructive mucosal leishmaniasis [1,2]. CL is the most frequent clinical form of ATL and is characterized by the presence of a skin ulcer, which heals spontaneously or after antimonial therapy [2,3]. While spontaneous healing appears to be associated with natural resistance, the immunological mechanisms of resistance have not been clearly defined. It was shown that early treatment fails to prevent ulcer formation in CL [4].

Despite the CD4^+^ T-cell-mediated immune response play a pivotal role in the processes either for cure or aggravation of the disease, some reports highlighted that CD8^+^ T lymphocytes may also play important role in the mechanisms for cure of and resistance to *Leishmania* infection [5-10]. Although the role of CD8^+^ T cells has been well established in these studies, there is a controversial statements about protective or deleterious function of effector subpopulation which has not been elucidated so far [7,11-17]. Previous researches have focused mostly on immune responses during active phase and at the clinical cure of disease, thus the investigation of immunological patterns of patients during the antimonial therapy is critical for better understanding the establishment of pathology and for determine beneficial parameters of the immune responses associated with clinical cure.

CD8^+^ T lymphocytes are functionally heterogeneous and the involvement of effector, *naïve* and memory CD8^+^ T-cell subsets has already been described in antitumor immune responses [18]. It is well established that human-effector CD8^+^ T cells have the CD45RA^+^CD27^−^ phenotype and these subset is thought to result from CD8^+^CD27^+^ precursors in response to antigenic stimulation [19-22]. To date there are few reports about the role of CD8^+^ T-cell subpopulations in the modulation of CL immune response and their functional activity should be better investigated.

Some authors have shown that apoptosis is involved in modulation of the immune response and may be directly related to the immunopathogenesis of some diseases including leishmaniasis [7,23-26]. Our previous results suggest that active disease and spontaneous cure of CL patients have been associated with higher or lower percentages of apoptotic CD8^+^ T cells, respectively [7]. Nevertheless, the association between apoptosis and functionally-defined CD8^+^ T-lymphocyte subsets in CL patients still remains undefined.

The present study investigates frequency and apoptosis of total and effector CD8^+^ T lymphocytes, in blood smears from CL patients before, during and after treatment, as well as evaluates antigen-specific effector CD8^+^ T-lymphocyte frequency and correlating immunological features with lesion size.

## Methods

### Study Groups

All CL patients enrolled in this study live in *Leishmania braziliensis*-endemic areas in Rio de Janeiro, Brazil [2] and were recruited at Leishmaniasis Surveillance Laboratory, Evandro Chagas Clinical Research Institute (IPEC), Oswaldo Cruz Foundation (FIOCRUZ), Rio de Janeiro, Brazil. All patients are volunteers and informed consent was obtained from all individuals prior to collection of blood samples. Diagnosis of leishmaniasis was based on clinical, laboratorial and epidemiological criteria. Ulcerated cutaneous lesions were associated with positive Montenegro skin test (MST) and positive parasitological exams to confirm a diagnosis of CL. All patients were submitted to meglumine antimoniate treatment according to the guidelines of the Brazilian Ministry of Health and sub-divided in three cohorts: Patients before treatment (PBT, n = 8, 36 ± 9 years old), evaluated after confirmed diagnosis and before beginning of anti-*Leishmania* treatment; patients during treatment (PDT, n = 14, 35.7 ± 13.4 years old), evaluated at the tenth day after beginning anti-*Leishmania* treatment, still showing ulcerated skin lesions; and patients after treatment (PAT, n = 11, 41 ± 15,19 years old), at the eighty day after the beginning of treatment. After treatment, all patients presented clinical cure, which was defined as full epithelialization of ulcerated lesions, regression of crusts, desquamation and infiltration. Healthy subjects (HS, n = 18, 29 ± 9.7 years old), from non-endemic areas, showing neither previous history of leishmaniasis nor any other co-morbidity, such as inflammatory diseases, diabetes or cardiologic disease, was analyzed similarly. The duration of lesion ranged from one month (less than 30 days) to six months and the larger diameter measured of the ulcers varied from 15 to 60 mm (PBT: 40 ± 5.3 mm; PDT: 42 ± 12.5 mm; PAT: 41.4 ± 13.9 mm). Basic demographic information of the studied groups is summarized in Table 1.

**Table 1:** **Demographic and clinical information of groups included in the study**

	**HS**	**PBT**	**PDT**	**PAT**
Number of volunteers	18	8	14	11
Sex: M/F	11/7	7/1	9/5	8/3
Age	29 ± 9.7	36.1 ± 9	35.71 ± 13.4	41 ± 15.1
Number of lesions	NA	1	1	1
Diameter of lesion (mm) (BF)	NA	40 ± 5.3	42 ± 12.5	41.4 ± 13.9
Montenegro Skin Test (MST) (mm) (BF)	NA	11.3 ± 1.8	11.7 ± 3.6	12.3 ± 3.6
Duration of disease (months)	NA	2 (1–6)	2 (1–6)	2 (1–5)

Age; Diameter of lesions; and MST: mean ± Standard Deviation.

Duration of disease: median (range).

BF = measured Before Treatment.

NA = Not Applicable.

### Ethics statement

This study was approved by National Ethical Clearance Committee of Brazil (CONEP) as well as by the Ethical Committee for Human Research from Oswaldo Cruz Foundation (CEP-FIOCRUZ) and Evandro Chagas Clinical Research Institute (CEP-IPEC/FIOCRUZ), Brazil. All of them adhere to the principles established in the Declaration of Helsinki on human subject research. Written informed consent was taken from all volunteers prior to blood collection.

### *Ex vivo* and *in vitro* phenotypic and apoptotic assays

Heparinized venous blood was obtained from CL patients and HS and peripheral blood mononuclear cells (PBMC) were obtained by Ficoll-Hypaque density gradient centrifugation (Sigma Aldrich, St. Louis, MO, USA) separation. A fraction of these cells was stained *ex vivo* and another was submitted to *in vitro* stimulation assay, where PBMC were adjusted (3x10^5^/well) in RPMI medium supplemented with 10% of AB Rh^+^ inactivated human serum (Sigma Aldrich) and then distributed in triplicate in a 96-well, flat-bottomed plate (Becton Dickinson, San Jose, CA, USA), as described previously [27]. Cells were stimulated with particulate antigens of *L. braziliensis* (LbAg) (disrupted in repeated freeze/thaw cycles and a final 5-minutes ultrasonication). Non-stimulated and 1 μg/well-concanavalin A (ConA)-stimulated cells (Sigma Aldrich) were used as negative and positive controls of proliferation, respectively. Cultures were carried out in a humidified atmosphere of 5% CO_2_ at 37°C. The time of incubation of ConA-stimulated cells was three days, while non-stimulated and LbAg-stimulated cells were five days. After that, cells were harvested and prepared for staining protocols.

### Cell surface and apoptosis staining protocol

Staining protocol was performed as previously described [7]. Briefly, *ex vivo* or *in vitro* assay’s cells were stained for surface markers with a panel of monoclonal antibodies, as follows: FITC-conjugated anti-CD3; APC-conjugated anti-CD8; PECy7-conjugated anti-CD27; ECD-conjugated anti-CD45 (all Beckman Coulter, Miami, FL, USA) in PBS containing 0.1% sodium azide (NaN_3_; Sigma Aldrich) and 2% fetal calf serum (Sigma Aldrich) and incubated for 20 minutes on ice. Afterwards, these samples were incubated with 20 μg/mL of 7-aminoactinomycin D (7-AAD; Sigma Aldrich) for 30 minutes at 4°C, for apoptosis evaluation, as described in elsewhere. The samples were kept on 7-AAD solution, protected from light, until the flow cytometry acquisition.

### Flow cytometry

**Figure 1:**
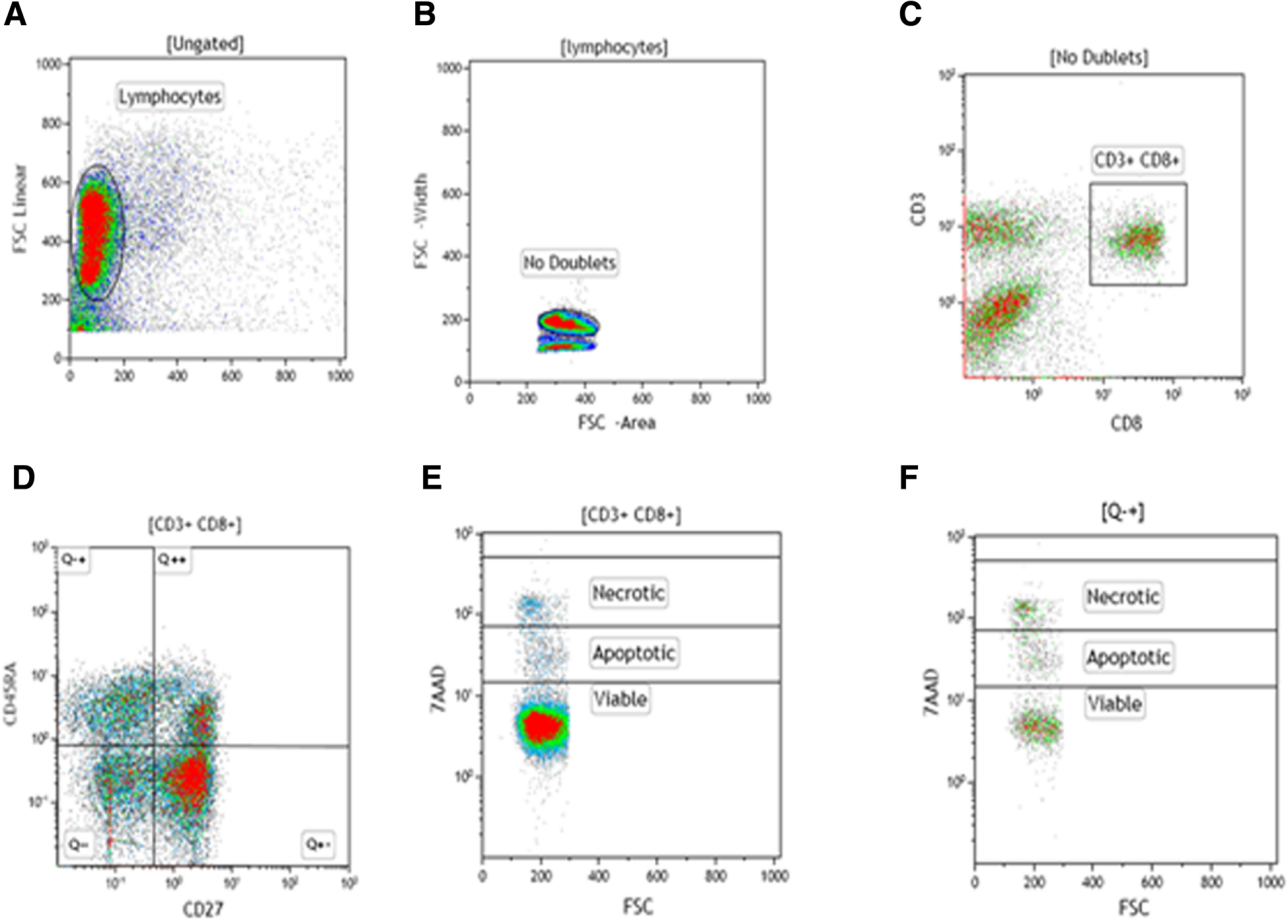
**Representative flow cytometry protocol to determine the frequencies of CD8** ^**+**^ **T-lymphocyte subsets and apoptosis.** Peripheral blood mononuclear cells (PBMC) from cutaneous leishmaniasis patients were stained *ex vivo* and after antigenic-stimulated cultures with CD3-FITC, CD8-APC, CD45RA-ECD, CD27-PE-Cy7 and 7-AAD. The lymphocytes were gated on forward (FSC) *vs* side (SSC) scatter dot-plot **(A)**, backgated from CD3^+^ histogram and doublets were excluded by a density plot of FSC Area *vs* FSC Width **(B).** CD27 *vs* CD45RA dot plot **(D)** gated on CD3^+^CD8^+^ region **(C**) was used to define the frequencies of effector and *naïve* CD8^+^ T lymphocytes. Frequency of apoptotic cells (7AAD^low^) was determined by FSC *vs* 7AAD dot-plot gated on CD3^+^CD8^+^ cells (total CD8^+^ T lymphocytes) **(E)** and on CD45RA^+^CD27^neg^ cells (effector CD8^+^ T lymphocytes) **(F)**.

Fifty thousand-event acquisitions were performed on Beckman Coulter Cyan ADP and on BD FACSAria II flow cytometers. The limits for the quadrant markers and histograms were always set based on non-staining cells and isotypic controls and color compensations were made based on simple labeling samples. A multiparameter flow cytometric protocol to determine the frequencies of total and effector CD8^+^ T lymphocytes and apoptosis was done in Kaluza 1.2 software (Beckman Coulter, Inc., Brea, CA, USA). In this manner, the frequency of total CD8^+^ T lymphocytes was determined in a CD3 vs. CD8 dot plot (Figure 1C) created from a region encompassing lymphocyte population in a SSC vs. FSC density plot (Figure 1A), excluding doublets (Figure 1B). To evaluate the frequency of effector CD8^+^ T-lymphocyte subsets a CD27 vs. CD45RA dot plot gated on CD3^+^CD8^+^ region was created and data CD27^+^CD45RA^−^ was recorded (Figure 1D; Q − +). Simultaneously, for apoptosis determination in total (Figure 1E) and effector (Figure 1F) CD8^+^ T cells a FSC vs. 7AAD dot plot was created gated on dot plots represented in Figure 1C and Figure 1D, respectively.

### Statistical analysis

For statistical analyses between two groups at a time, Mann–Whitney *U* test was used. For comparison between nonstimulated and stimulated CD8^+^ T-lymphocyte subsets, we used a paired nonparametric Wilcoxon test. These results were reported as mean ± standard error (SEM). We also used a Spearman’s rank correlation test. Correlations and intergroup differences were considered statistically significant when *P <* 0.05. All statistical calculations and graphical representations of data were obtained using the GraphPad Prism version 5.0 software (GraphPad Software Inc., La Jolla, CA, USA).

## Results

### Frequency of total and effector CD8^+^ T cells

**Figure 2:**
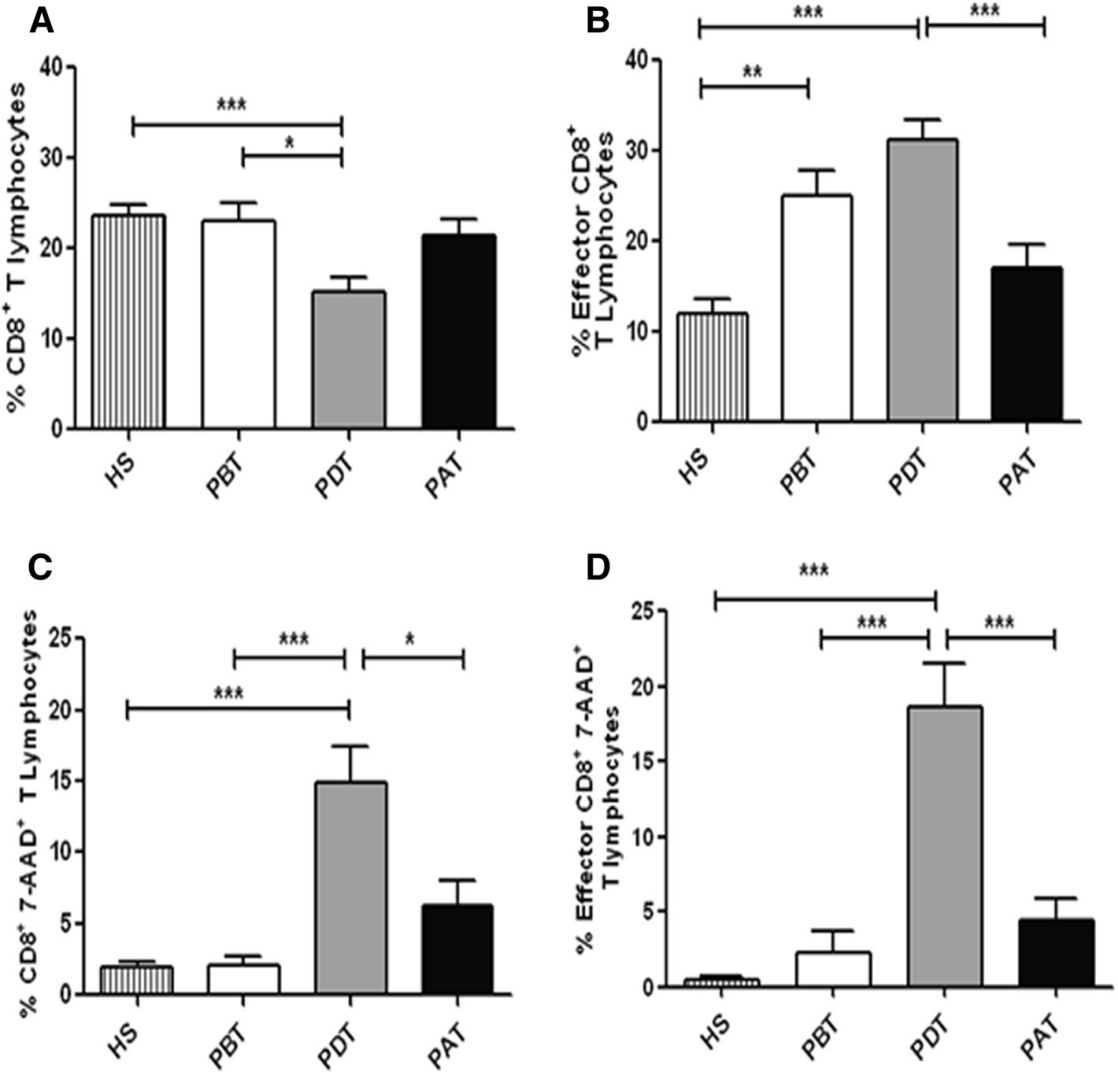
***Ex vivo*** **analysis of total CD8** ^**+**^ **T-lymphocyte frequency and apoptosis in human cutaneous leishmaniasis. (A)** Total CD8^+^ T lymphocytes; **(B)** Effector CD8^+^ T lymphocytes; **(C)** Apoptotic-total CD8^+^ T lymphocytes; **(D)** Apoptotic-effector CD8^+^ T lymphocytes. HS - healthy subjects (n = 18); PBT – patients before treatment (n = 8); PDT - patients during treatment (n = 14); PAT - patients after treatment (n = 11). Statistical analyses were performed by Mann Whitney Test. The bars represent the mean ± standard error. Results were considered significant with *P* < 0.05 - ^*^(*P <* 0.05) **(*P <* 0.01) ***(*P* < 0.001).

We performed comparative analysis of the frequency of total CD8^+^ T cells from patients before treatment (PBT), patients during treatment (PDT) and patients after treatment (PAT), as well healthy subjects (HS). The mean frequency of total CD8^+^ T cells was significantly lower in PDT (15.3 ± 1.5) compared to PBT (23 ± 2; *P* < 0.05) and to HS (23.6 ± 1.2; *P <* 0.001). These lower frequency also were seen when comparing PDT with in PAT (21.5 ± 1.6; *P* = 0.07), although these difference was not statistically significant (Figure 2A).

Due to the heterogeneity of the peripheral CD8^+^ T-cell pool, we performed a dichotomized analysis in order to discriminate the differential distribution of their subsets. Thus, in order to highlight the importance of effector CD8^+^ T lymphocytes in the parasitic immune responses, we analyzed the CD8^+^CD45RA^+^CD27^−^ T cells, an effector phenotype. We observed a higher frequency of these cells in PDT (31.2 ± 2.2) compared to other three groups, HS (12 ± 1.4; *P <* 0.001), PBT (25 ± 2.7; *P* < 0.05) and PAT (16.9 ± 2.7; *P <* 0.001) (Figure 2B). PBT also showed higher frequency of these cells when compared to HS and PAT. It is important to note that PBT and PDT showed lower percentages of CD8^+^CD45RA^+^CD27^+^*naïve* T-cell subset when compared to HS and to PAT, which could be a consequence of differentiation of *naïve* in effector CD8^+^ T cells, during active disease (data not shown).

### Apoptosis of total and effector CD8^+^ T cells

Previous report of our group showed that there was a higher rate of apoptotic-total CD8^+^-T lymphocytes in non-healing lesions of CL when compared to lesions that progress to spontaneous cure [7], suggesting a modulate role of apoptosis on these cells in CL lesion environment. Following this hypothesis, we investigated the role of apoptosis in blood compartment, through the 7-AAD staining and flow cytometry. The results of e*x vivo* analyses showed higher frequencies of apoptotic-total (14.9 ± 2.8) and apoptotic-effector CD8^+^ T cells from PDT (18.6 ± 2.8) when compared to: PBT (apoptotic-total, 2 ± 0.6; *P* < 0.001; apoptotic-effector, 2.3 ± 1.4; *P* < 0.001); PAT (apoptotic-total, 6.2 ± 1.7; *P* < 0.05; apoptotic-effector, 4.3 ± 1.5; *P* < 0.001); and HS (apoptotic-total, 1.8 ± 0.4; *P* < 0.001; apoptotic-effector, 0.4 ± 0.2; *P <* 0.001) (Figure 2C and D). These results showed pronounced percentages of apoptotic CD8^+^ T lymphocytes only on patients during treatment, which tend to decrease after the end of treatment indicating that this phenomenon could be associated to the glucantime therapy and the immune response triggering.

### *Leishmania braziliensis*-reactive CD8^+^ T lymphocytes

**Figure 3:**
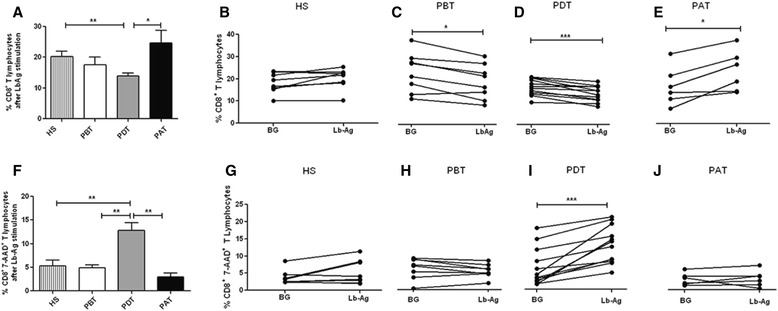
***In vitro*** **analyses of total (A – E) and apoptotic (F – J) CD8** ^**+**^ **T-lymphocyte frequency. (A and F):** comparison among the percentages of *Leishmania braziliensis* antigen (LbAg)-stimulated cells from HS - healthy subjects (n = 8); PBT – patients before treatment (n = 8); PDT - patients during treatment (n = 11); and PAT - patients after treatment (n = 6). Statistical analyses were performed by Mann Whitney Test and the bars represent the mean ± standard error. **(B, C, D, E, G, H, I, J):** comparison between stimulated (Lb-Ag) and nonstimulated cells (BG – background) from HS; PBT; PDT and PAT, respectively. Solid lines connect the results for the same individual. Statistical analyses were performed by paired, nonparametric Wilcoxon test. Results were considered significant with *P <* 0.05. ^*^(*P <* 0.05) **(*P <* 0.01) ***(*P <* 0.001).

In order to determine an expansion of CD8^+^ T cells involved in a specific anti-Leishmania T-cell response, PBMC were cultured in the absence and in the presence of *L. braziliensis* antigens (LbAg). Frequencies of LbAg-reactive-total CD8^+^ T cells were compared among the four studied groups. PDT showed lower mean frequencies of *Leishmania braziliensis*-reactive CD8^+^ T lymphocytes (13.8 ± 1.0) when compared to PAT (24.7 ± 3.9; *P <* 0.05) (Figure 3A); to HS (20.2 ± 1.6; *P* < 0.01); and to PBT (17.5 ± 2.5), although the difference between the frequencies of PBT and PDT was not statistically significant (*P* = 0.2) (Figure 3A). To evaluate the modulation in the frequencies of LbAg-reactive CD8^+^-T cells, we performed paired analyses between the percentage of nonstimulated-total CD8^+^ T cells (background - BG) and those of LbAg-stimulated CD8^+^ T cells. Both PBT (BG, 21.4 ± 2.8; LbAg, 17.5 ± 2.5; *P <* 0.05) and PDT (BG, 16.9 ± 1; LbAg, 13.8 ± 1; *P* < 0.01) showed lower frequencies of LbAg-reactive total CD8^+^ T cells, more pronounced in PDT (Figure 3C and D, respectively). On the other hand, PAT showed a higher frequency of these cells (BG – 17.7 ± 3.7; LbAg – 24.7 ± 3.9; *P <* 0.05) (Figure 3E), showing that LbAg down-modulate CD8^+^ T cells during *in vitro* assays with cells obtained from PBT and PDT. In the opposite manner, LbAg up-modulate these cells in assays performed with cells from patients after treatment and clinical cure. No changes on frequency of these cells were seen in experiments with cells from HS (BG, 18.1 ± 1.6; LbAg, 20.2 ± 1.6) (Figure 3B).

Corroborating the results showed in *ex vivo* findings, we observed higher rates of apoptotic-CD8^+^ T cell in cultures with LbAg-stimulated cells from PDT (12.8 ± 1.5) compared to PBT (4.9 ± 0.6; *P* < 0.01); PAT (2.9 ± 0.8; *P <* 0.01); and HS (5.2 ± 1.2; *P* < 0.001) (Figure 3F). The paired analysis confirmed that the higher frequency of apoptotic-total CD8^+^ T lymphocytes from PDT (BG, 6.4 ± 1.5; LbAg, 12.8 ± 1.5; *P* < 0.001) is antigen-dependent (Figure 3I), and could not be seen in HS- (BG, 3.7 ± 0.7; LbAg, 5.2 ± 1.2), in PBT- (BG, 5.6 ± 0.9; LbAg, 4.9 ± 0.6) neither in PAT-*in vitro* experiments (BG, 2.7 ± 0.6; LbAg, 2.9 ± 0.8) (Figure 3G, H and J, respectively).

**Figure 4:**
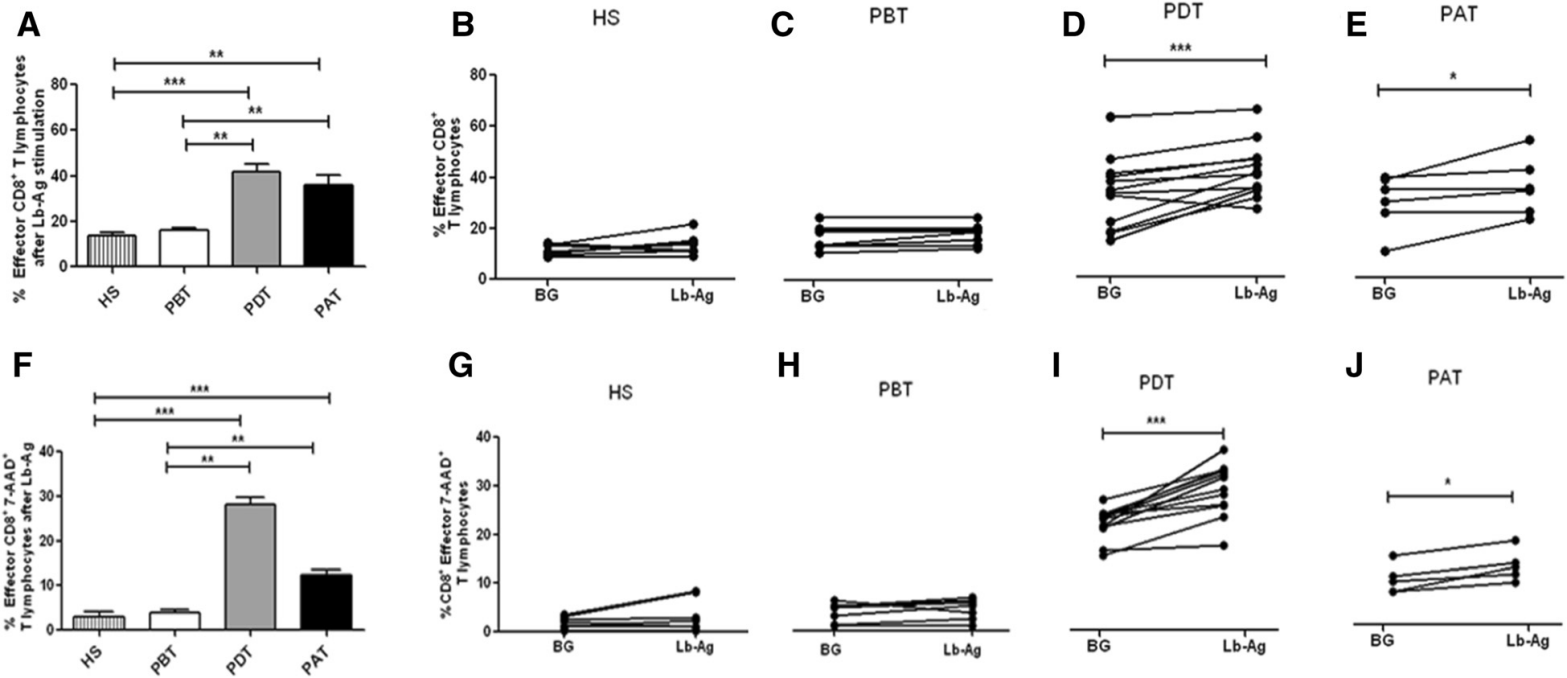
***In vitro*** **analysis of effector (A – E) and apoptotic-effector (F - J) CD8** ^**+**^ **T-lymphocyte frequencies. (A and F)**: comparison among the percentages of *Leishmania braziliensis* antigen (LbAg)-stimulated cells from HS - healthy subjects (n = 8); PBT – patients before treatment (n = 8); PDT - patients during treatment (n = 12); and PAT - patients after treatment (n = 6). Statistical analyses were performed by Mann Whitney Test and the bars represent the mean ± standard error. **(B, C, D, E, G, H, I, J)**: comparison between antigen-stimulated (Lb-Ag) and nonstimulated cells (BG – background) from HS; PBT; PDT and PAT, respectively. Solid lines connect the results for the same individual. Statistical analyses were performed by paired, nonparametric Wilcoxon test. Results were considered significant with *P <* 0.05. ^*^(*P <* 0.05) **(*P <* 0.01) ***(*P <* 0.001).

Regarding frequency of LbAg-reactive-effector CD8^+^ T cells during *in vitro* assays, it was observed higher frequencies of these cells in experiments with cells from PDT (41.8 ± 3.1) and from PAT (35.8 ± 4.6) when compared to experiments with cells from PBT (15.9 ± 1.3; *P <* 0.01) and also to HS (13.4 ± 1.3; *P <* 0.01 and *P* < 0.001) (Figure 4A). The modulation of frequency of these cells in the presence of these antigens was confirmed by the paired test in which we detected higher frequencies in PDT (BG, 33.1 ± 4; LbAg, 41.8 ± 3.1; *P <* 0.001) and PAT (BG, 29.8 ± 4.3; LbAg, 35.8 ± 4.6; *P <* 0.05) (Figure 4D and E, respectively), while PBT (BG, 14.6 ± 1.5; LbAg, 15.9 ± 1.3; *P <* 0.01) and HS showed similar frequencies among stimulated and nonstimulated cells (BG, 11.3 ± 0.7; LbAg, 13.4 ± 1.3) (Figure 4C and B, respectively).

Concerning the apoptotic-effector CD8^+^ T cells, a comparison among the four studied groups showed higher percentages in PDT (28.1 ± 1.5) when compared to HS (3 ± 1.1, *P* < 0.001) and PBT (3.8 ± 0.7, *P <* 0.01). We also observed higher frequencies in PAT (12.3 ± 1.1) when compared to HS (*P* < 0.001) and PBT (*P <* 0.01) (Figure 4F). The paired analysis have shown significant differences of apoptotic-effector CD8^+^ T cells between BG and LbAg in PDT (BG, 21.2 ± 0.9; LbAg, 28.1 ± 1.5; *P* < 0.001) and PAT (BG, 9.6 ± 1; LbAg, 12.3 ± 1.1; *P* < 0.05) (Figure 4I and J, respectively), while no significant difference was observed in HS (BG, 1.7 ± 0.4; LbAg, 3 ± 1.1) and PBT (BG, 3.2 ± 0.7; LbAg, 3.8 ± 0.7) (Figure 4G and Figure 4H, respectively).

### Correlation analysis of effector and apoptotic-effector CD8^+^ T lymphocytes with lesion size

**Figure 5:**
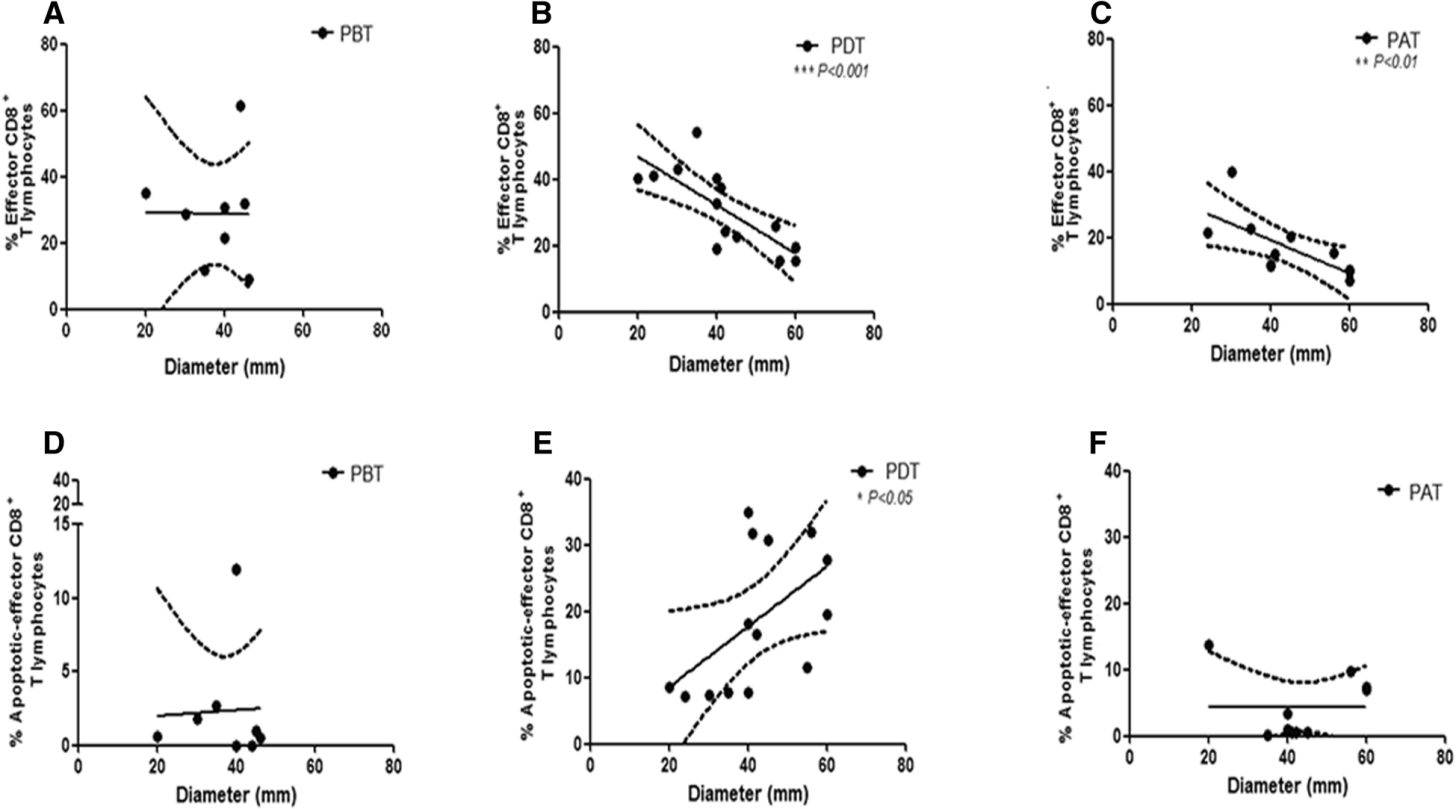
**Clinical characteristics correlated with immunological parameters from cutaneous leishmaniasis patients.** Correlation between the percentage of effector CD8^+^ T-lymphocytes **(A, B, C)** and apoptotic-effector CD8^+^ T-lymphocytes **(D, E, F)** with diameter of lesion (mm). **(A, D)** PBT - patients before treatment (n = 8); **(B, E)** PDT – patients during treatment (n = 14); **(C, F)** PAT - patients after treatment (n = 11). The graphics show fit lines with confidence curves. Statistical analyses were performed by Spearman’s correlation test. Results were considered significant with *P <* 0.05.

Taking account the relationship between clinical features and immune response in CL, we correlated the frequencies of effector and apoptotic-effector CD8^+^ T lymphocytes with lesion size. Results showed an inverse correlation between frequencies of effector CD8^+^ T lymphocytes and lesion size in PDT (r = −0.79; *P* < 0.001) as well as in PAT (r = −0.79; *P* < 0.01). The lower the frequency of effector CD8^+^ T cells, the larger the size of lesion (Figure 5B and C, respectively). In contrary, no statistical correlation was observed between the frequencies of effector CD8^+^ T lymphocytes and lesion size in PBT (Figure 5A). These results suggest that a greater induction of effector CD8^+^ T cells after the beginning of treatment would be associated with small lesions, less inflammatory process and minor tissue destruction. Correlation analyses between lesion size and antigen-specific CD8^+^ T cell were done but no statistically significant result could be observed (data not shown).

Earlier report of our group showed high frequencies of apoptotic-total CD8^+^ T cells in lesions of patients with active CL compared with patients evolved to spontaneous cure [7]. Hence, became noteworthy to correlate the frequencies of circulating-apoptotic-effector CD8^+^ T cells to lesion sizes in order to identify a T cell subset implicated either protective or deleterious role during the treatment or after clinical cure of CL. It was observed a positive correlation between higher frequencies of apoptotic-effector CD8^+^ T cells and larger lesion areas in PDT (r = 0.62; *P <* 0.05) (Figure 5E), although there were no correlation between these parameters in PBT and in PAT (Figure 5D and C, respectively).

## Discussion

The host immune response to *Leishmania* is mainly mediated by T cells [28]. The study of immunopathogenesis in human CL has been based on the determination of the frequency of CD4^+^ and CD8^+^ T lymphocytes and cytokine production [13,27,29]. Earlier observations from our group have shown that CD8^+^ T lymphocytes have a role in the cure process in CL patients [7,8,10,27,30] and other reports reinforces this hypothesis [14,31]. Conversely, some authors have associated the CD8^+^ T lymphocytes to tissue injury in CL [15] and in mucocutaneous leishmaniasis [11,12]. It is important to note that, in all of these reports, patients were evaluated before and after antimonial therapy not taking into account the immunological events that happen during treatment. In order to assess the characteristics of the immune response involved in the healing process of CL patients, it is of utmost importance the evaluation of patients during antimonial therapy. Our results showed important differences in the CD8^+^ T-cell frequencies, characterizing early and final phases of clinical cure, which seems to be linked to antimonial therapy.

The frequency of apoptotic CD8^+^ T cells in HS is in accordance to the normal apoptotic rate (1–4%) as reported elsewhere [32]. Because PDT showed higher frequencies of apoptotic CD8^+^ T cells than PBT and HS, we suggest that apoptosis of these cells could be related to the beginning of therapy. The highest frequency of apoptotic CD8^+^ T lymphocytes observed during the antimonial treatment could be associated to lower rate of total CD8^+^ T cells in PDT, suggesting an association between apoptosis and a down-modulation of the total CD8^+^ T cells. It is in accordance with a previous report of our group, which have shown that high rates of apoptotic-total CD8^+^ T cells was related to active disease, while a lower frequency of apoptotic-total CD8^+^ T cells is related to spontaneous cure [7]. Brelaz et al. [27] related the key role of CD8^+^ T cells in the process of healing with a significantly higher proportion of circulating CD8^+^ T lymphocytes in spontaneously healed patients when compared to patients before treatment. Elevated frequencies of total CD8^+^ T cells in PAT compared to PDT, may represent a tendency of these cells to reestablish levels of normality at the end of treatment and could be associated to clinical cure.

We observed an increase of apoptotic-total CD8^+^ T cells and a decrease of total CD8^+^ T-cell frequencies in LbAg-stimulated cultures with cells from PDT as well as from PBT. Inversely we observed an increase of total CD8^+^ T-cell frequencies in LbAg-stimulated cultures with cells from PAT, showing that at the end of treatment, total CD8^+^ T lymphocytes could expand in response to LbAg. Based in these findings we may hypothesize that the high rates of apoptosis observed in *ex vivo* total CD8^+^ T cells from PDT could be triggered by expressive amount of circulating antigen derived from parasite destruction caused by the antimony.

The high frequency of LbAg-reactive total CD8^+^ T lymphocytes observed after therapy corroborates data found by Da-Cruz et al. [10,23] who suggested that the increased levels of these cells at the end of treatment would be associated with resolution of lesion. It is worth to note that these studies compared patients before and after treatment and there was a need to supplement this information, evaluating patients during treatment. So, the increased levels of LbAg-reactive total CD8^+^ T lymphocytes observed at the end of therapy indicate that probably an expansion of this cell population was not perceived at early phases of healing process. It is in accordance with others [33,34] who reported a later development of an efficient immune response, i.e., when there is a balanced response with control of parasite replication without tissue injury.

Despite the knowledge about the key role of CD8^+^ T lymphocytes in the immune response, the evaluation of effector CD8^+^ T-cell subset became an imperative approach for better understanding the specific role of these cells, in healing process in patients under treatment [19,20,35]. Besides, the relationship between frequencies of effector CD8^+^ T cells and the different stages of treatment is unknown. Taking into account that effector CD8^+^ T lymphocytes represent 10 to 40% of total-circulating CD8^+^ T cells and this pool includes a variety of functionally distinct subpopulations, an analysis of effector population can provide information about some functional characteristics of this subset, which would be imperceptible when the analysis of total CD8^+^ T lymphocytes was performed.

Concerning *ex vivo* analysis of effector CD8^+^ T cell, the highest percentage observed in PDT seems to indicate a greater induction of this subset during the treatment. Considering that Glucantime® is a leishmanicidal drug, a higher amount of circulating antigen during treatment might induce effector CD8^+^ T lymphocytes and explain the higher frequency of these cells in PDT. Some reports corroborates this statement, as demonstrated by Meymandi et al. [36] where clearly showed that, at their histological findings, there was a reduction in aggregations of histiocytes, decreased cellular parasitic load and an important increased numbers of CD3^+^ T cells in response to combining antimonial treatment. In another study there was an increase in the percentage of CD8^+^ T cells in peripheral blood from patients with leishmaniasis under treatment with meglumine antimoniate [37]. Moreover, the lower frequency of CD8^+^ T cells observed in PAT may be related to a reduced antigenic stimulation, which could depict what is happening *in vivo* after clinical cure.

Because effector CD8^+^ T lymphocytes from PDT and PAT expanded in response to LbAg while PBT did not, our study indicated that antimonial treatment might not influence the involvement of effector CD8^+^ T lymphocytes in the antigen-specific immune response to parasite.

Apoptosis is a physiological process of immune responses, however this phenomenon of death can also be a modulating factor of immunopathogenesis of some disorders such as Dengue, Chagas’ disease and AIDS [21,31,32]. In the present report, high apoptosis rates observed in LbAg-stimulated effector CD8^+^ T cells in PDT point to the occurrence of activation-induced cell death (AICD), suggesting that this death phenomenon may be happening *in vivo* during treatment [33]. Although PAT showed low frequencies of effector CD8^+^ T lymphocytes, when compared to PDT, the small rates of apoptotic-effector CD8^+^ T lymphocytes seem to favor to clinical cure. Moreover, in parasitic infections the cross-talk between apoptosis of T lymphocytes and cytokine production was associated to a deleterious role of this death phenomenon [26].

Some authors considered that the severity of disease could be characterized by lesion size, which is considered as the most significant clinical feature in CL [38]. Herein, we showed that the smaller the size of lesion, the greater the frequency of effector CD8^+^ T lymphocytes in PDT and PAT. Some authors [34] reported that the size of lesion found in patients evaluated before therapy was directly related to activated T lymphocytes. Thus, we may postulate that after the beginning of antimonial therapy there is a greater induction of effector CD8^+^ T cells, which is inversely proportional to the lesion size, suggesting that a small frequency of effector CD8^+^ T lymphocytes can favor to the tissue damage. Agreeing this data, we showed that the greater the size of lesion, the higher the frequency of apoptotic-effector CD8^+^ T lymphocytes in PDT, suggesting a deleterious role of this death phenomenon. This observation corroborates with our previous report [7] where we observed that patients evolved to spontaneous cure were associated to small frequencies of apoptotic CD8^+^ T lymphocytes. This data emphasize the protective role of CD8^+^ T cells considering the severity of lesions. Further studies are underway to determine what functional characteristics these effector cells have, since they can present cytotoxic and/or pro-inflammatory-cytokine-producer profiles.

## Conclusions

Taking together, our results showed an evident expansion of effector CD8^+^ T lymphocytes in response to *LbAg*, more pronounced in PDT. Changes in effector CD8^+^ T–lymphocyte frequencies, during and after treatment, seem to represent a critical stage to generate an efficient immune response and suggest that these cells would be evolved in the triggering or in the resolution of lesion, when under the influence of therapy. Although this work do not define the effective role of CD8^+^ T cells in the CL immunopathogenesis, our findings put forth the notion that the evolution to cure induced by antimonial therapy implicate the effector CD8^+^ T lymphocytes. Moreover, our results emphasize the protective role of CD8^+^ T cells considering the severity of lesions. Further studies are underway to determine what functional characteristics these effector cells have, since they can present cytotoxic and/or pro-inflammatory-cytokine-producer profiles. This new approach of evaluating patients during treatment proved to be very important for understanding the healing process. Furthermore, this report might be used as a basis for further investigations concerning antimonial therapy and to guide vaccine investigations based on the development of an effective cellular immune response that regulates tissue damage in human cutaneous leishmaniasis.

## Abbreviations

7-AAD: 7 aminoactinomycin D
AICD: Activated-induced cell death
AIDS: Acquired immunodeficiency syndrome
APC: Allophycocyanin
ATL: American tegumentary leishmaniasis
BD: Becton & Dickinson
BF: Measured before treatment
BG: Background
CD: Cluster of differentiation
CL: Cutaneous leishmaniasis
ConA: Concanavalin A
CONEP: National Ethical Clearance Committee of Brazil
ECD: Energy couple dye
FIOCRUZ: Oswaldo Cruz Foundation
FSC: Forward scatter
FITC: Fluorescein Isothiocyanate
HS: Health subjects
IPEC: Evandro Chagas Clinical Research Institute
LbAg: *Leishmania braziliensis* antigen
MST: Montenegro skin test
NA: Not applicable
PAT: Patients after treatment
PBMC: Peripheral blood mononuclear cells
PBT: Patients before treatment
PDT: Patients during treatment
PECy7: Phycoerythrin Cyanin 7
SSC: Side scatter
